# Etiological stratification and prognostic assessment of haemophagocytic lymphohistiocytosis by machine learning on onco-mNGS data and clinical data

**DOI:** 10.3389/fimmu.2024.1390298

**Published:** 2024-09-09

**Authors:** Lin Wu, Xuefang Cao, Jingshi Wang, Qi Kong, Junxia Hu, Lin Shi, Liurui Dou, Deli Song, Leilei Chen, Mengyuan Zhou, Huan Liu, Ruotong Ren, Zhao Wang

**Affiliations:** ^1^ Department of Haematology, Beijing Friendship Hospital, Capital Medical University, Beijing, China; ^2^ Research and Development (R&D) Department, MatriDx Biotechnology Co., Ltd., Hangzhou, China; ^3^ Research and Development (R&D) Department, EBV-Care Biotechnology Co., Ltd., Beijing, China; ^4^ Research and Development (R&D) Department, Micro-Health Biotechnology Co., Ltd., Beijing, China; ^5^ Foshan branch, Institute of Biophysics, Chinese Academy of Sciences, Beijing, China

**Keywords:** hemophagocytic lymphohistiocytosis, etiological stratification, prognostic assessment, onco-mNGS, machine learning

## Abstract

**Introduction:**

Hemophagocytic lymphohistiocytosis (HLH) is a rare, complicated and life threatening hyperinflammatory syndrome that maybe triggered by various infectious agents, malignancies and rheumatologic disorders. Early diagnosis and identification of the cause is essential to initiate appropriate treatment and improve the quality of life and survival of patients. The recently developed Onco-mNGS technology can be successfully used for simultaneous detection of infections and tumors.

**Methods:**

In the present study, 92 patients with clinically confirmed HLH were etiologically subtyped for infection, tumor and autoimmunity based on CNV and microbial data generated by Onco-mNGS technology, and a predictive model was developed and validated for the differential diagnosis of the underlying disease leading to secondary HLH. Furthermore, the treatment outcomes of patients with HLH triggered by EBV infection and non-EBV infection were evaluated, respectively.

**Results:**

The current study demonstrated that the novel Onco-mNGS can identify the infection and malignancy- related triggers among patients with secondary HLH. A random forest classification model based on CNV profile, infectious pathogen spectrum and blood microbial community was developed to better identify the different HLH subtypes and determine the underlying triggers. The prognosis for treatment of HLH patients is not only associated with CNV, but also with the presence of pathogens and non- pathogens in peripheral blood. Higher CNV burden along with frequent deletions on chromosome 19, higher pathogen burden and lower non-pathogenic microbes were prognosis factors that significantly related with unfavorable treatment outcomes.

**Discussion:**

Our study provided comprehensive knowledge in the triggers and prognostic predictors of patients with secondary HLH, which may help early diagnosis and appropriate targeted therapy, thus improving the survival and prognosis of the patients.

## Introduction

Hemophagocytic lymphohistiocytosis (HLH), also known as a hemophagocytic syndrome, is a life-threatening hyperinflammatory syndrome caused by aberrantly activation of cytotoxic T lymphocytes, natural killer cells, and macrophages, resulting in hypercytokinemia and immune-mediated injury in multiple organs (1). As a syndrome, the clinical manifestations of HLH vary, including fever, organomegaly, cytopenia, consumptive coagulopathy, hypertriglyceridemia, and elevation of acute-phase reactants. Subspecialists in different fields, such as hematology/oncology, infectious diseases, rheumatology/clinical immunology are challenged by this rare multifaceted syndrome since patients often suffer from recurrent fever, cytopenia, liver dysfunction, and a sepsis-like syndrome that may rapidly progress to terminal multiple organ failure. Therefore, although HLH is a rare disease, clinical physicians should be aware of HLH, because early recognition may prevent irreversible organ damage and subsequent death (2, 3).

HLH is generally classified as primary (genetic) or secondary (reactive), in which primary HLH is the predominant subtype in children and the secondary HLH is the most common type in adults patients (1, 4). Secondary HLH is commonly triggered by infections or malignancies, but also be induced by autoimmune disease and medications (5). Although HLH can occur at any age, but most clinical guidelines, prospective studies, and treatment trials have focused on paediatric patients. The treatment protocols HLH-94 and HLH-2004 have been established as scientific cornerstones for diagnosis, classification, and treatment of HLH in patients younger than 18 years (6, 7). In the year 2019, the HLH Steering Committee of the Histiocyte Society developed these recommendations for diagnosis and treatment of HLH in adults (1). Infections are the most prevalent triggers of HLH, and a variety of infective pathogens are associated with HLH, mainly viruses such as *Epstein-Barr virus* (EBV) and *Cytomegalovirus* (CMV), but also bacteria, parasites, and fungi. It is reported that viral infection is the most frequent trigger, either as a primary infection in healthy people or after reactivation in immunosuppressed patients, of which *Herpes viruses* account for 62% of reported viral cases of HLH in adults, and 43% of the viral cases are due to EBV and 9% to CMV (4). Bacterial infections are reported in 9% of adult HLH cases, of which 38% were due to tuberculosis. Parasites and fungi are rare triggers of HLH, with *histoplasma*, *leishmania*, *plasmodium*, and *toxoplasma* being the most frequently reported. Though the list of infections that have been reported to occur with HLH is extensive, some cases are influenced by geographic region (leishmaniasis and tick-borne illnesses), season (influenza viruses, tick-borne illnesses), and socioeconomic status (tuberculosis). Adult HLH has also been associated with a variety of malignancies, including T-cell or natural killer cell lymphomas, B-cell lymphomas, leukemias, Hodgkin lymphoma, other hematologic neoplasms, solid tumors, and other non-specified neoplasms (4). Besides, HLH can also occur during chemotherapy and is often associated with an infection (8).

A comprehensive evaluation and systematic diagnosis of patients with suspected HLH is required due to the complex etiology of HLH. In the clinical practice, several simultaneous assessments should begin once the patient was suspected with HLH. Laboratory and imaging studies should be performed to gather supportive evidence of a diagnosis of the syndrome of HLH and assess which organ systems are involved and the severity of involvement, and evaluations for infections and malignancies should be performed in all patients, including laboratory studies, bone marrow evaluation, general imaging, and biopsy of any suspicious findings (9). As HLH is an aggressive and fatal syndrome, early diagnosis and identification of the cause are essential to initiate appropriate treatment and improve the quality of life and survival of patients. However rapid identification of the underlying infectious cause of HLH is challenging because traditional etiological diagnostics are time-consuming and sometimes fail to identify the pathogens (10). Besides, traditional diagnosis of malignant tumors is also time-consuming with a low positive rate, and it greatly increases the psychological and economic burden on patients due to invasive measures. The newly emerging metagenomic next-generation sequencing (mNGS) may be a potential optimal solution, which may help improve the clinical diagnosis of underlying infections in hematological diseases. Owing to its unbiased, rapid and broad-range detection capability, mNGS has been applied in many complicated infection cases related HLH and successfully identified the underlying pathogenic microorganisms (11–19). However, conventional mNGS was only designed for rapid identification of pathogens associated with infectious diseases. Previous studies have demonstrated that the recently developed Onco-mNGS technology can be successfully used for simultaneous detection of infections and tumors (20–22). This is because it not only allows for the precise identification of pathogenic microorganisms in clinical samples based on microbial data from mNGS, but also provides information on human chromosome copy number variations (CNVs) in patients with malignancy by analysis of human-derived genomic data (23, 24). Nevertheless, the clinical value of Onco-mNGS technology for HLH patients remains to be further explored.

Herein, we attempted to evaluate the diagnostic performance of Onco-mNGS in patients with HLH to further improve the efficiency of HLH etiologic screening. In the present study, 92 patients with clinically confirmed HLH were etiologically subtyped for infection, tumor and autoimmunity based on CNVs and microbial data generated by Onco-mNGS technology, and a predictive model was developed and validated for the differential diagnosis of the underlying disease leading to secondary HLH. Furthermore, the treatment outcomes of patients with HLH triggered by EBV infection and non-EBV infection were evaluated, respectively.

## Methods

### Study design and patients

**Figure 1:**
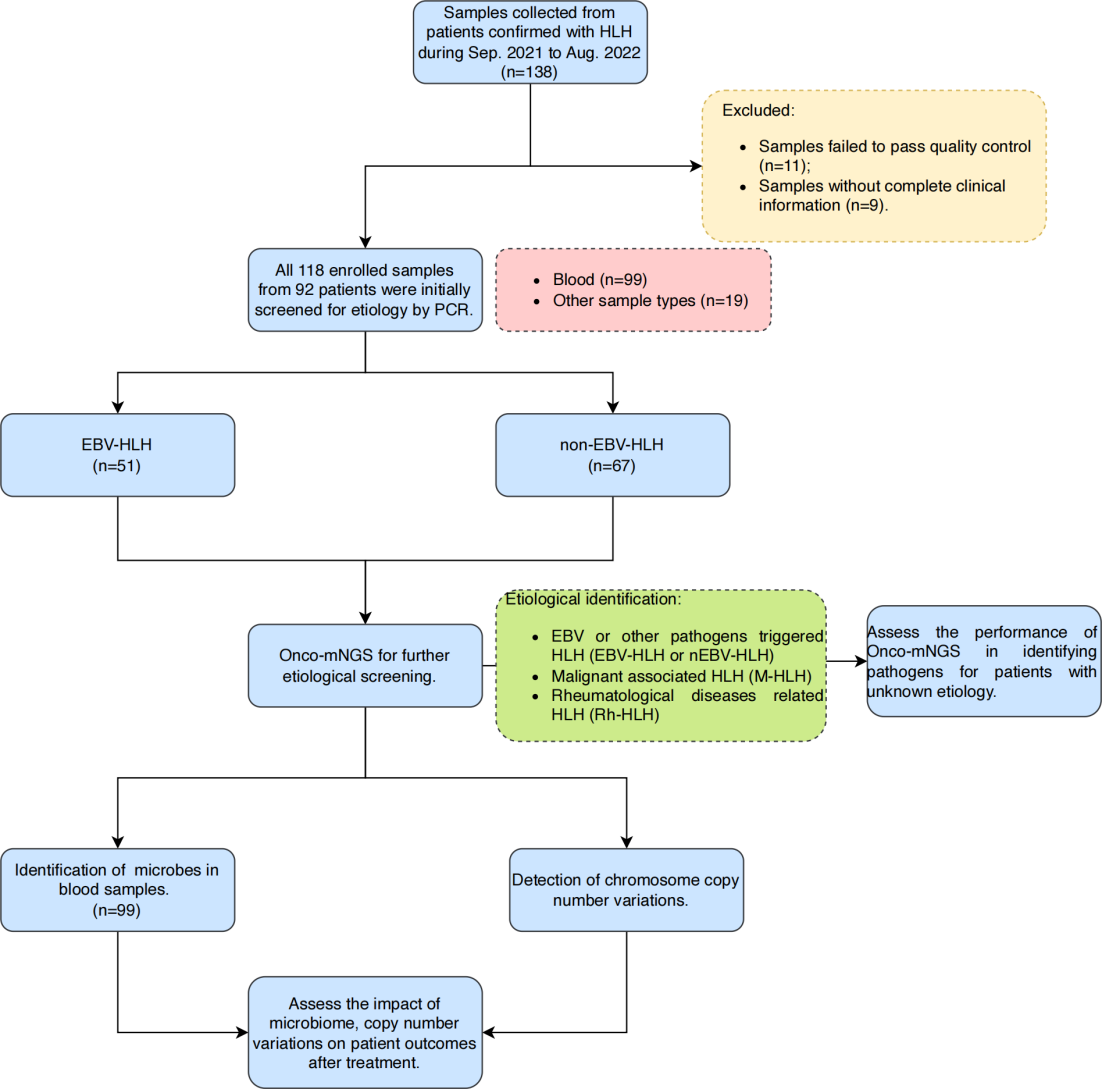
Overall design and flowchart of this study. A total of 118 samples were finally enrolled in this study after excluding samples that failed pass the quality control and without complete clinical information. Blood samples were selected for further microbes identification and chromosomal copy number variations analysis in this study. Abbreviations: HLH, hemophagocytic lymphohistiocytosis; mNGS, metagenomic next-generation sequencing.

The present study was conducted using the remaining samples routinely collected at Department of Haematology, Beijing Friendship Hospital, Capital Medical University between September 2021 and August 2022 from patients diagnosed with HLH based on HLH-2004 diagnostic criteria (**Figure 1**). Since the causative factors of secondary HLH are similar in both paediatric and adult populations (mainly complex infections, tumours and autoimmune diseases, etc.), and since there is an urgent need for studies related to stratified diagnosis and prognostic assessment in both paediatric and adult patient populations, the age range of the patients included in the present study was 1-76 years (with a median age of 35 years), with a male/female ratio of 44.57%:55.43% (**Table 1**). As a single-centre retrospective clinical study, we firstly completed the random enrolment of patients based on the definitive diagnosis of secondary HLH, then completed the clinical grouping of the etiology of each patient with secondary HLH after clinical diagnostic data retrieval and clinical confirmation, and further completed the Onco-mNGS testing of peripheral blood samples of each patient. Finally, based on the CNV parameters and characteristic microorganisms obtained and combined with the clinical indicators, we completed the construction and efficacy assessment of random forest binary or ternary classifiers for secondary HLH etiological stratification diagnosis and treatment prognosis assessment.

**Table 1:** Patient and sample characteristics.

Characteristics	Value
Patient demographics (n = 92)	
Age (years)	
Median (IQR)	35 (25.5-52)
Range	1-76
Gender, n (%)	
Female	41 (44.57)
Male	51 (55.43)
Clinical characteristics, n (%)	
EBV infected IC-HLH	43 (46.74)
non-EBV infected IC-HLH	25 (27.17)
M-HLH	13 (14.13)
Rh-HLH	11 (11.96)
Treatment, n (%)	
On antibiotics treatment	78 (84.78)
Treatment outcomes	
CR/PR^a^	70 (76.09)
NR^b^	22 (23.91)
Sample characteristics (n = 118)	
Sample type, n (%)	
Peripheral blood	99 (83.90)
Sputum	11 (9.32)
Pleural fluid	3 (2.54)
BALF^c^	2 (1.69)
CSF^d^	2 (1.69)
Bone marrow	1 (0.85)

a CR/PR, Complete remission/Partial remission.

b NR, non-remission.

c BALF, bronchoalveolar lavage fluid.

d CSF, cerebrospinal fluid.

The study was approved by the Ethics Committee of Beijing Friendship Hospital, Capital Medical University, and all data were anonymized prior to analysis. The study was conducted in accordance with the Declaration of Helsinki and the study data were obtained from Department of Haematology, Beijing Friendship Hospital, Capital Medical University. Informed consent was obtained from all participants or their legal guardians.

### DNA extraction, library preparation and NGS

Body fluid samples and other types of specimens from the residual specimens in the clinical laboratory were collected. DNA extraction and library preparation from clinical samples were performed by using an NGS Automatic Library Preparation System (MatriDx Biotech Corp. Hangzhou). The quality of DNAs was assessed using a BioAnalyzer 2100 (Agilent Technologies; Santa Clara, CA, United States) combined with quantitative PCR to measure the adapters before sequencing. The libraries were then adjusted (aiming for 20 million (M) reads) and pooled for NGS on an Illumina NextSeq550Dx system using the sequencing strategy of single-end (SE) 50 base pairs (bp). For contamination control, irrelevant cell line-based control samples were used in parallel throughout the process.

### Pathogen determination and analysis of abnormal CNV signatures

A total of 10-20 million reads were obtained for each sample. Clean reads obtained after raw data demultiplexing and adapter trimming were subjected to microbial identification based on a reference database containing over 20000 microorganisms. All microorganisms detected in clinical samples based on Onco-mNGS are first filtered with those detected in the parallel NTC (no template control) (background microorganisms), and remained microbes with a ratio of unique reads per million (RPM) above 10 or if the organism was not detected in the parallel NTC. All the microorganisms authentically present in clinical samples were defined as microbiota. Substantially, all species of microbiota were looked up in PubMed to determine whether the organisms cause infection, and the positive pathogenic microorganisms were defined as pathogens.

Simultaneously, sequencing reads were mapped to the human reference genome (hg19) from the NCBI database, and only uniquely positioned reads were selected for subsequent analysis. The reference genome was fragmented into contiguous windows of fixed length, and read depths were calculated for each window and then normalized to the total number of reads per sample. The copy number ratio for each window was obtained by dividing the normalized read depth by the average read depth in the reference dataset. The copy number was then taken as log2 and adjacent windows with similar ratios are combined into fragments annotated with chromosome position and average ratio. The copy number of each fragment was calculated based on the mean ratio and normal copy number of the corresponding chromosome and then compared to a preset threshold to validate the CNV.

The results of the etiological screening of the enrolled patients were evaluated by a panel of clinical experts (including three experienced physicians). Onco-mNGS results were interpreted according to MatriDx Biotechnology Co., Ltd.’s own pathogen data filtering principles. Infectious diseases are diagnosed based on microbiological tests, Onco-mNGS results and clinical review results. Tumors are judged based on Onco-mNGS results in addition to histopathology, cytological examination and microscopic examination and other validation tests.

### Treatments

Treatment strategies were based on the HLH-94/04 protocol, and included dexamethasone, etoposide, and cyclosporine (7, 25). Treatment response and dynamic changes during the 8 weeks of treatment were evaluated. Complete response was defined as resolution of all clinical signs and symptoms, CBC recovery, and normalization of abnormal laboratory findings associated with HLH. Partial response was defined as either CBC recovery or normalization of laboratory findings. Progressive disease was defined as persistence of cytopenia and abnormal laboratory findings. No response means those whose disease continues to progress, die or without any response during therapy. Treatment responses were then divided into two groups, the remission group with complete and partial response, the non-remission group with no response.

### Statistical analysis

All collected data were statistically analyzed using R packages. Categorical variables, shown as frequencies and percentages, were compared using Fisher’s exact test. Continuous measurement data following normal distribution were shown as mean (standard deviation) or mean (standard error), and non-normal distribution was shown by median (range). Differences and significance between groups were calculated using Student’s t-test (for a normal distribution data) and Wilcoxon rank sum test or Kruskal-Walli’s test (for non-normal distribution data). Data visualization was performed in R (Version 4.2.3). Alpha diversity indices were characterized by Shannon, Richness, Simpson and Inverse Simpson. The t-test was used to assess the differences among groups. Beta diversity was described by Unconstrained PCoA (principal coordinate analysis) and the Analysis of similarities (Anosim) was used to examine differences between groups. Linear discriminant analysis effect size (LEfSe) analysis was conducted to determine microorganisms that were significantly different in abundance between groups, with thresholds of log10 LDA (linear discriminant analysis) Score≥2 and *P* value ≤ 0.05. RPM values of microbes were log-transformed before their relative abundance was analyzed. In this study, two-sided *P* values < 0.05 were considered statistically significant. The random forest method was used to construct a predictive model to assess whether the specific microbes and clinical indicators could be used as biomarkers to distinguish different patient groups, using ten-fold cross-validation. The predictive performance of the classifiers was analyzed by ROC curves.

## Results

### Demographics and clinical characteristics of patients

**Figure 2:**
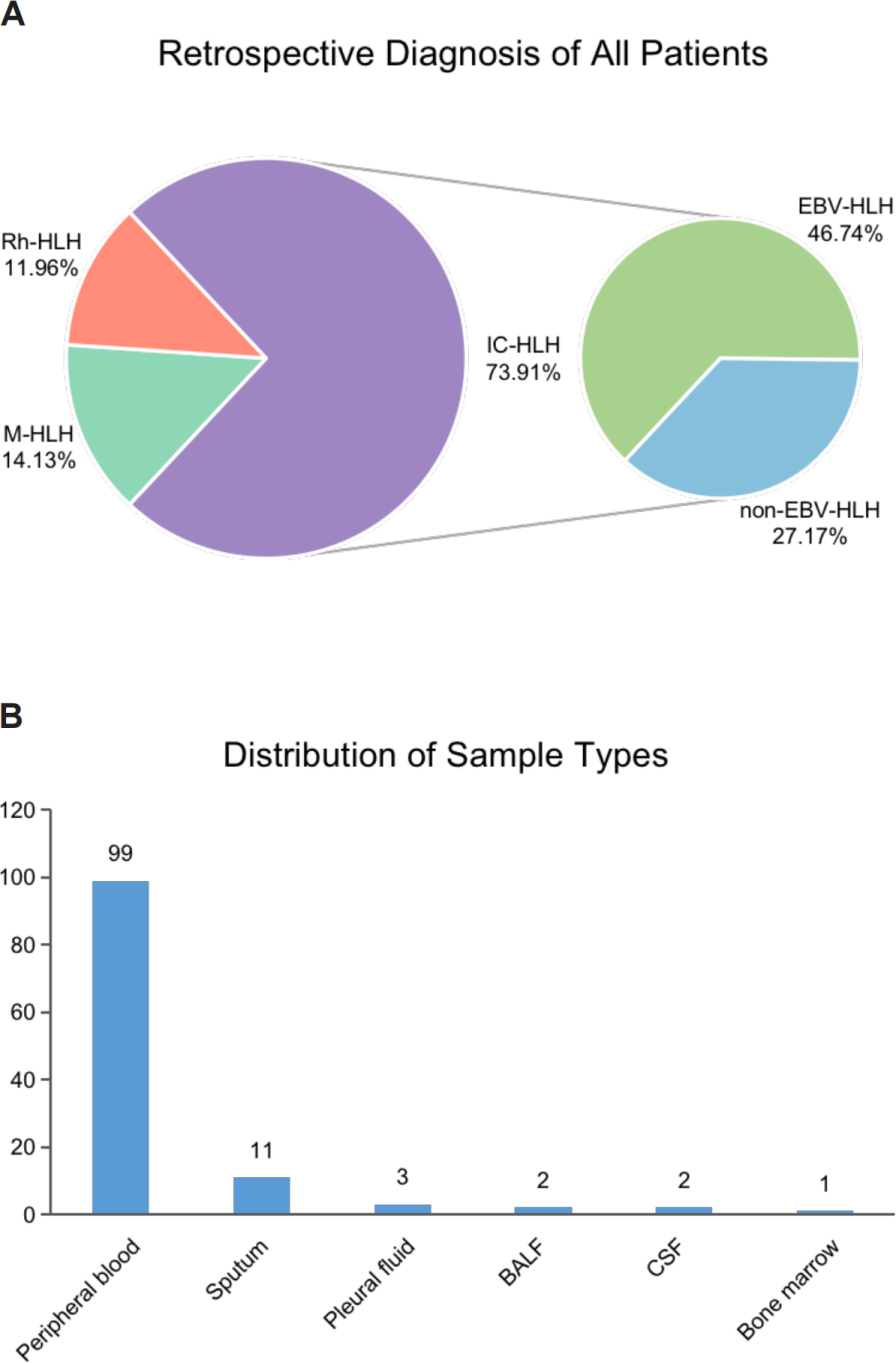
The distribution of HLH subtypes and clinical specimens in the present study. **(A)** Retrospective diagnosis of all patients. **(B)** Distribution of sample types. Abbreviations: BALF, bronchoalveolar lavage fluid; CSF, cerebrospinal fluid.

A total of 138 body fluid samples from 92 patients were collected between September 2021 and August 2022 from Beijing Friendship Hospital, Capital Medical University (**Figure 1**). After excluding samples that failed pass the quality control and without complete clinical information, 118 samples were finally enrolled in this study. Clinical characteristics of all patients and samples enrolled are summarized in **Table 1**. The median age of the enrolled patients was 35 years old, however the age distribution was not uniform, ranging from 1 to 76 years old. Most patients (84.78%) underwent antibiotic treatment during sample collection. About 76% of patients received improved outcomes (CR/PR), while the rest had no remission (23.91%). All the patients were diagnosed with HLH based on HLH-2004 diagnostic criteria, and divided into four subtypes, including EBV (n=43, 46.74%) or other pathogen (n=25, 27.17%) triggered HLH (EBV-HLH and nEBV-HLH), and malignant (n=13, 14.13%) or rheumatologic disorders (n=11, 11.96%) associated HLH (M-HLH and Rh-HLH) (**Figures 1**, **2A**). Body fluid samples from the residual specimens in the clinical laboratory were collected, the majority of which are blood samples (n=99) (**Figure 2B**). Other sample types included sputum (n=11), pleural fluid (n=3), BALF (n=2), CSF (n=2) and bone marrow (n=1). For further analysis, Onco-mNGS was performed using all blood samples for microbe identification, meanwhile the sequences of human DNA yield from Onco-mNGS were used for chromosomal copy number analysis.

### Differentiation of EBV-HLH and M-HLH to other HLH subtypes by random forest analysis on CNV characteristics

**Figure 3:**
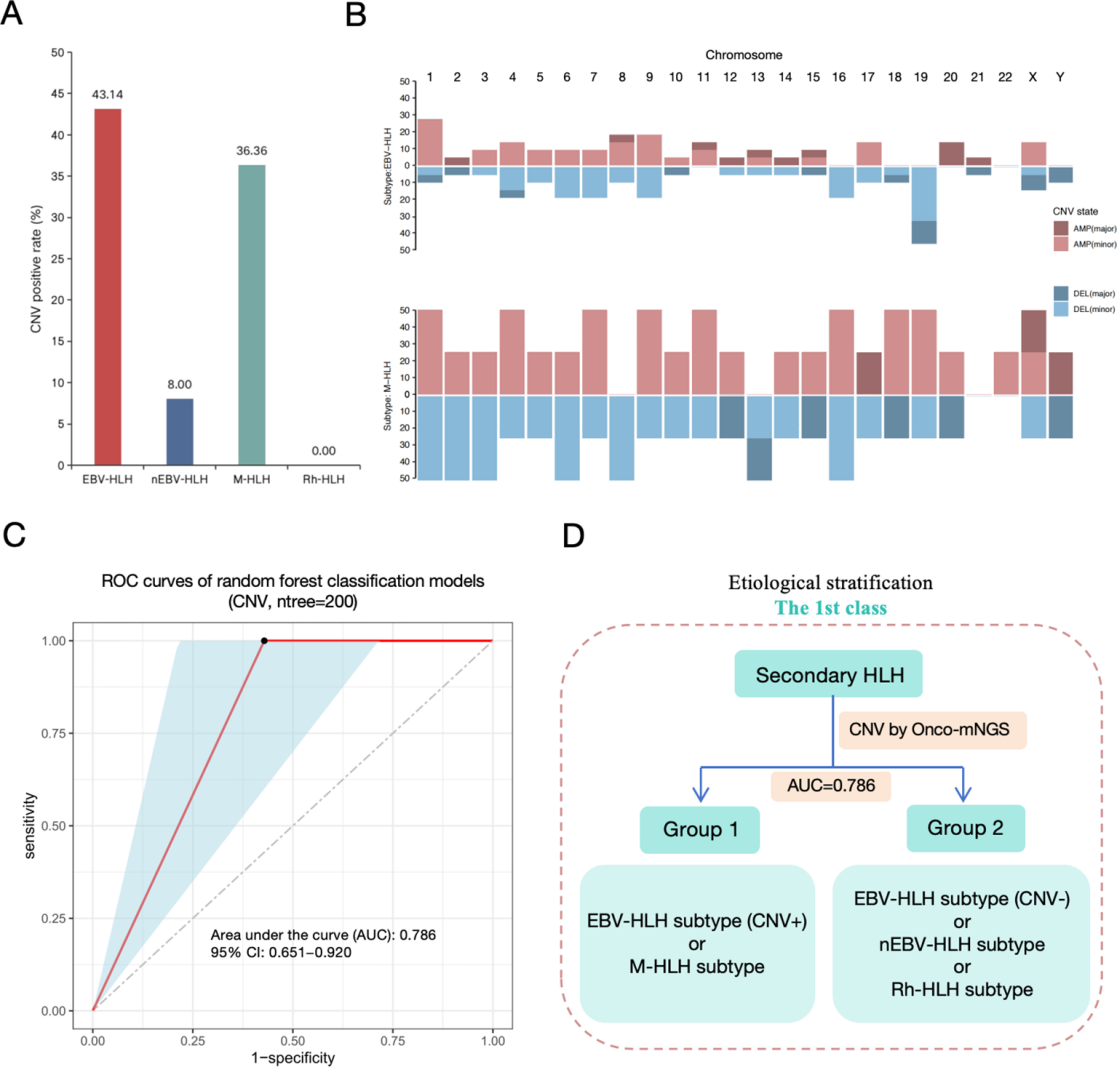
First-level etiological stratification of secondary HLH diagnosis based on analysis of CNV data derived from Onco-mNGS. **(A)** Copy number variation (CNV) positive rate detected by mNGS. **(B)** Distribution and frequncy of gain (AMP, amplification) or loss (DEL, deletion) of DNA segments in subtype EBV-HLH and subtype M-HLH. **(C)** The efficacy assessment of a diagnostic model for the etiological stratification of secondary HLH at the first level based on CNV data (ROC curve). **(D)** Diagram of the first level of etiological stratification for the diagnosis of secondary HLH. The Random Forest binary classifier constructed on the basis of CNV data could better distinguish Group 1 (CNV-positive EBV-HLH and M-HLH) from Group 2 (CNV-negative EBV-HLH, nEBV-HLH, and Rh-HLH), with an AUC value of 0.786.

The sequences of human DNA derived from mNGS were used for chromosomal copy number analysis. The positive rate of CNV occurrence in each subtype was analyzed in **Figure 3A**. Two subtypes emerge to be with the most unstable genome, which are EBV-HLH (43.14%) and M-HLH (36.36%) subtypes. Both CNV amplification and deletion were observed in different HLH subtypes. However, the distribution of CNV on different chromosomes are distinct in EBV-HLH subtype and M-HLH (**Figure 3B**). M-HLH subtype exhibited relatively high incidence of CNV on all chromosomes except chromosome 21, whereas CNV on chromosome 19 was the most common genomic events in EBV-HLH subtype. M-HLH subtype and EBV-HLH subtype also differed in CNV types on some chromosomes. On chromosome 8 and 13, only CNV deletion occurred in M-HLH subtype, whereas both CNV deletion and duplication occurred in EBV-HLH subtype. The reverse result was observed on chromosomes 16, 18 and 19. In addition, the frequency of CNV deletions on chromosome 19 was observed to be more than 40% in EBV-HLH, compared with 25% in M-HLH subtype. Based on CNV parameters, a random forest model was constructed to classify two groups including Group 1 (partial subtype EBV-HLH and subgroup M-HLH) and Group 2 (partial subtype EBV-HLH, subtype nEBV-HLH and subtype Rh-HLH) as the first level of aetiologically stratified diagnosis for secondary HLH. The training and testing sets were 3:1, and ten-fold cross-validation methods were performed to determine the number of significant variables for classification. As shown in **Figure 3C**, we accessed the diagnostic performance of the classifiers by ROC curves and the current classifier displayed satisfying diagnostic performances in typing subtypes EBV-HLH plus M-HLH and subtypes nEBV-HLH plus Rh-HLH, with an average AUC of 0.768. To sum up, a hierarchical diagnostic schematic was drawn for illustrating that Group 1 and Group 2 can be better distinguished using the CNV-based Random Forest Biclassifier model (**Figure 3D**).

### Signatures of pathogens identified in patients diagnosed with different HLH subtypes by onco-mNGS

A total of 18 pathogens were detected in all blood samples, including 11 viruses, 4 bacteria and 3 fungi species (**Supplementary Figure 1A**). Two kind of viruses, *Human gammaherpesvirus 4* and *Human betaherpesvirus 5*, were the most common species identified in all samples. Besides, *Staphylococcus aureus* in the most frequent bacterial species. Most samples were identified with at least one pathogen based on Onco-mNGS sequencing. Among the EBV subtypes, relatively high pathogen burdens were observed in each sample. The stacked bar charts in **Supplementary Figure 1B** presented the relative abundance of all pathogens in different subtypes, as well as the dominated pathogenic species in each subtype. As expected, *Human gammaherpesvirus 4* was the most abundant pathogens in EBV-HLH subtype, which barely detected in nEBV-HLH and Rh-HLH subtypes. While *Human betaherpesvirus 5* commonly existed among three HLH subtypes besides EBV-HLH subtype. Besides the dominated *Human betaherpesvirus 5*, *Human alphaherpesvirus 1* and *Torque teno virus* were also commonly found in nEBV-HLH subtype, *Human gammaherpesvirus 4* and *Human betaherpesvirus 6B* were also the dominated pathogens in M-HLH subtype, and *Human alphaherpesvirus 3* was also commonly found in Rh-HLH subtype. Details of pathogen composition in each sample collected from patient with different HLH subtypes were shown in **Supplementary Figures 2A-D**.

Among four subtypes of HLH, the two subtypes triggered by pathogen infection, EBV-HLH and nEBV-HLH, showed relatively higher positive rate of mNGS sequencing, which complied with clinical consensus (**Supplementary Figure 3A**). In the present study, there are 144 microbial species were identified by mNGS sequencing in blood samples of HLH patients, consisting of 79% of bacteria, 11% of fungi and 10% of viruses (**Supplementary Figure 3B**). But for further analysis, clinical physicians were involved and performed adjudication on species identified by mNGS sequencing, aiming to separate the pathogens from non-pathogenic microbes. Evaluation the diagnostic performance of Onco-mNGS in identifying pathogens for patients with unknown etiology were also conducted. Taking the gold standard methods as comparison, the sensitivity of mNGS to identify EBV-HLH and nEBV-HLH subtype was 86.7% and 100% respectively (**Supplementary Figure 3C**). Positive rate and burden of the two frequent pathogens (*Human gammaherpesvirus 4* and *Human betaherpesvirus 5*) in blood from patients with different HLH subtypes were further illustrated in **Supplementary Figures 4A, B**. In EBV-HLH subtype, the positive rate of *Human gammaherpesvirus 4* was as high as 88.24%, and the pathogen burden was significantly higher than that of other subtypes (*P*<0.001). *Human betaherpesvirus 5* was found a highest positive rate in nEBV-HLH subtype, but the pathogen burden in each subtype showed no significant difference (*P*>0.05).

### Etiological stratification of secondary HLH based on microbiological data derived from onco-mNGS and patient clinical data

**Figure 4:**
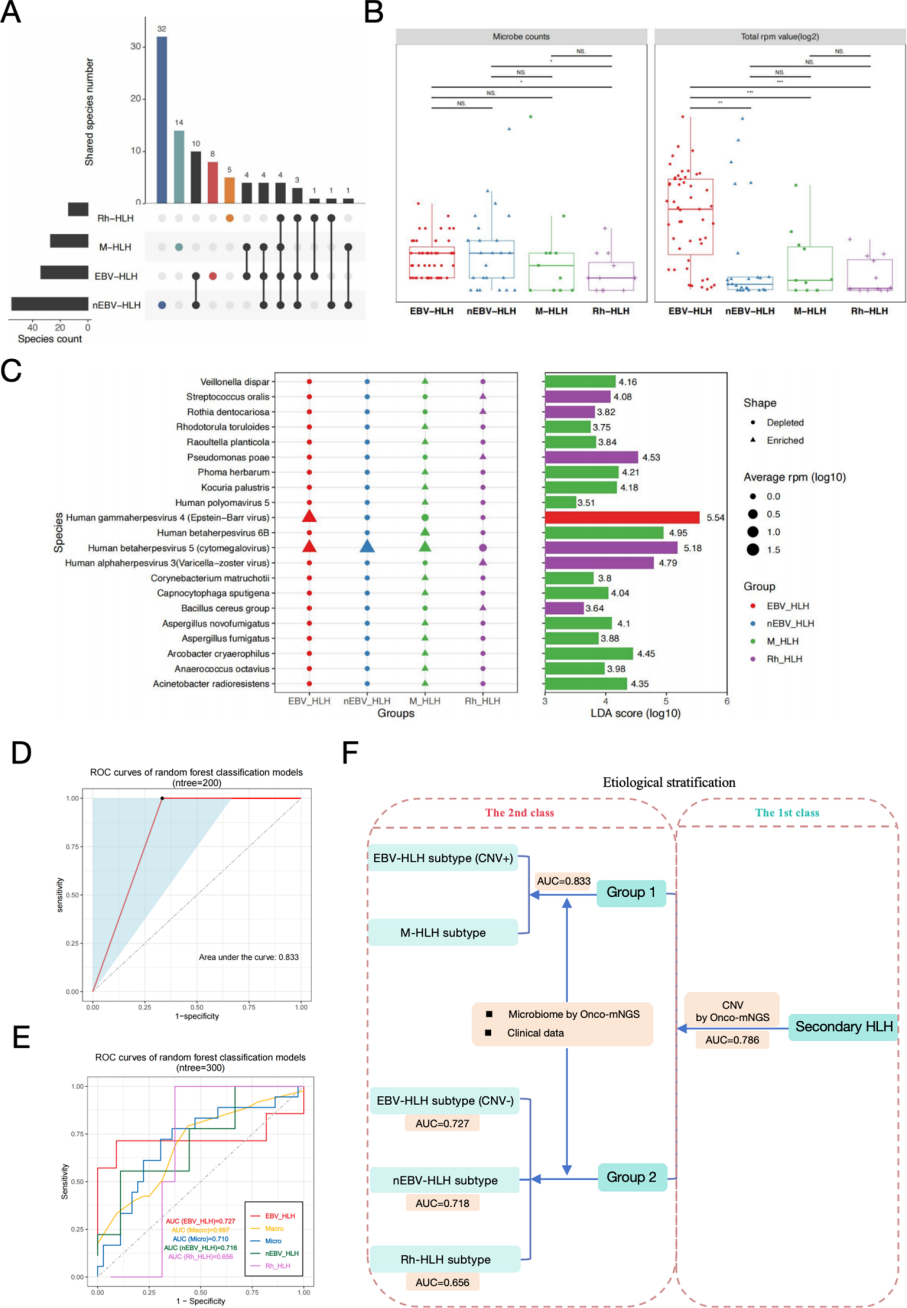
Secondary-level etiological stratification of secondary HLH diagnosis based on analysis of microbiome data derived from Onco-mNGS and clinical examination data. **(A)** Upset plots of blood microorganisms with frequencies above 3% in each HLH subtype identified by mNGS. **(B)** Box plots of blood microbial burdens in different HLH subtypes, differences between groups were assessed by T-test. **(C)** Specific blood microbial biomarkers in different subtypes were determined through LEfSe analysis. The microbial species enriched in the EBV-HLH, M-HLH and Rh-HLH subtypes were presented in the plot with respective average RPM value and LDA scores. Alpha value for the factorial Kruskal-Wallis test among classes was 0.01, and for the pairwise Wilcoxon test between subclasses was 0.05. A threshold value of 2.0 was applied to the log LDA score for discriminatory features. Significant differences between groups are indicated by asterisks, with * represents *P*<0.05, ** represents *P*<0.01, *** represents *P*<0.001. Abbreviations: PCoA, principal coordinate analysis; PERMANOVA, permutational multivariate analysis of variation; LEfSe, linear discriminant analysis effect size; LDA, linear discriminant analysis; NS., no significant difference. **(D)** The efficacy assessment of a diagnostic model for the etiological stratification of Group 1 (CNV-positive EBV-HLH and M-HLH) at the secondary level based on microbiome and clinical examination data (ROC curve). **(E)** The efficacy assessment of a diagnostic model for the etiological stratification of Group 2 (CNV-negative EBV-HLH, nEBV-HLH, and Rh-HLH) at the secondary level based on microbiome and clinical examination data (ROC curve). **(F)** Diagram of both the first level and the secondary level of etiological stratification for the diagnosis of secondary HLH.Two random forest binary classifiers and one ternary classifier constructed based on different types of data can better distinguish CNV-positive EBV-HLH, M-HLH, CNV-negative EBV-HLH, nEBV-HLH, and Rh-HLH in different hierarchical order effectively.

Microecology is increasingly considered to be involved in the onset and progression of diseases, therefore the diversity and dynamics of the microbial community for each subtype of HLH would suggest different etiologic mechanisms. Few species appeared to commonly exist among different subtypes, with only 4 shared microbes observed among all subtypes. nEBV-HLH subtype emerged to have the most complex and diverse microbial community with the most microbes identified, of which 32 species were uniquely existed (**Figure 4A**). All microbes in each sample were counted, and their corresponding RPM values detected by mNGS were summarized to verify the complexity among HLH subtypes at general level (**Figure 4B**). The microbe counts of two infection-induced subtypes, EBV-HLH and nEBV-HLH, were significantly increased in comparison with Rh-HLH subtype (*P*<0.05), but had no significance in comparison with M-HLH subtype. Additionally, the total RPM value of all microbes within each sample of EBV-HLH were significantly increased compared with other subtypes (*P* < 0.01). The general landscape of four HLH subtypes were summarized by heatmap in **Supplementary Figure 5A**. To compare the overall composition of the blood microbial signature in different HLH subtypes, the diversity within each subtype and between subtypes was analyzed by alpha-diversity and beta-diversity (**Supplementary Figures 5B, C**). Four indices were analyzed in order to demonstrate the alpha-diversity within each subtype, and results indicated that nEBV-HLH and M-HLH subtypes showed increased diversity, as determined by analysis of variance (*P*<0.05). Unconstrained PCoA (for principal coordinates PCoA1 and PCoA2) with Bray-Curtis distance showed that microbes detected in EBV-HLH subtype were separated from other HLH subtypes in the first axis (*P*<0.05), indicating the unique microbial community composition of EBV-HLH subtype. To further explore the differences in microbial species between different HLH subtypes, the Linear discriminant analysis Effect Size (LEfSe) method was performed to identify microbes that significantly enriched in each subtype. A total of 21 species were screened as differentially enriched microbes among HLH subtypes, most of which were found significantly abundant in M-HLH subtype (**Figure 4C**). Of these differential species, several species such as *Streptococcus oralis*, *Rothia dentocariosa*, *Pseudomonas poae*, *Human alphaherpesvirus 3*, *Human betaherpesvirus 5* and *Bacillus cereus group* were enriched in Rh-HLH subtype, but only *Human gammaherpesvirus 4* was significantly enriched in EBV-HLH subtype. Interestingly, the average RPM of *Human betaherpesvirus 5* in Rh-HLH were found lower than the other three subtypes, thus being classified as a depleted microbe. These different microbes provide evidence for a unique microbial community composition of different HLH subtypes.

After the above microbiome analysis, we obtained information about the characteristic microorganisms of different subgroups. Next, we used the characteristic microbial information and the clinical examination data to construct random forest classifier models for distinguishing the CNV-positive EBV-HLH subgroup and the M-HLH subgroup in Group 1 and the CNV-negative EBV-HLH subgroup, the nEBV-HLH subgroup and Rh-HLH subgroup. The ratio of the training set to the test set was 3:1, and the number of important variables for classification was determined using the tenfold cross-validation method. As shown in **Figure 4D**, we determined the diagnostic performance of the binary classifier by the ROC curve, and found that the binary classifier was able to effectively discriminate between the CNV-positive EBV-HLH subgroups and M-HLH subgroups in Group 1, with an AUC value of 0.833. Meanwhile, a random forest multivariate classifier constructed based on the characteristic microbial information and clinical examination data could effectively distinguish the CNV-negative EBV-HLH subgroup, the nEBV-HLH subgroup and the Rh-HLH subgroup in Group 2, with AUC values of 0.727, 0.718 and 0.656, respectively (**Figure 4E**). Finally, we drew a schematic diagram of secondary HLH stratified diagnosis containing the first and second levels, further illustrating that on the basis of the first level of stratified diagnosis based on CNV, the present study can continue to use the biomarker collections such as characteristic microbial information of different subgroups and prophylactic data to construct random forest binary classifier or multiclassifier models used for the second level of etiological stratified diagnosis (**Figure 4F**).

### The predictive assessment of treatment prognosis in EBV-HLH patients based on both CNVs and blood microbiome characteristics

**Figure 5:**
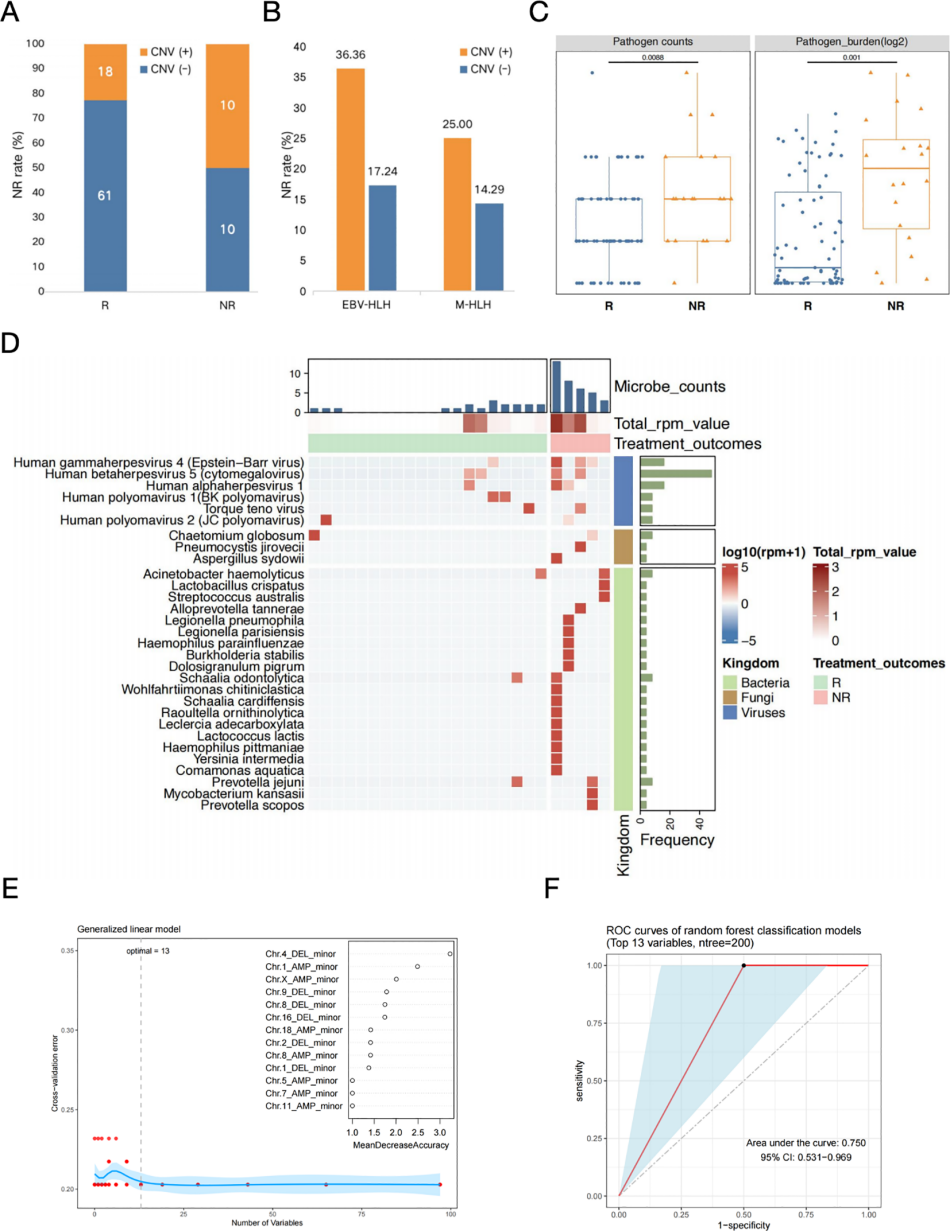
The prognosis prediction in EBV-HLH patients based on both CNVs and blood microbiome characteristics. **(A)** Occurrence of CNVs in patients with different treatment outcomes. **(B)** Non-remission rates in CNV positive or negative patients with different HLH subtypes. **(C)** Comparison of the counts and burdens of pathogens in HLH patients with different treatment outcomes. Differences between groups were assessed using T-test. **(D)** Heatmap of selected microbes with frequencies above 10% in each group (R and NR) identified by mNGS in patients with nEBV-HLH subtype. Microbes with frequency more than 10% within each group (R and NR) were selected, log10-transformed RPM of selected microbes were applied. Samples were hierarchically clustered within each group using Pearson correlation as a distance measure with average-linkage. Abbreviations: R, remission; NR, non-remission. **(E)** The determination of the number of significant variables for classification. The optimal point of cross-validation error determined by the number of biomarkers was 13, which implies that based on the mean decreasing accuracy, the top 13 variables which are all CNV-related parameters could be selected as potential markers used to differentiate between secondary HLH treatment effects (R vs. NR). **(F)** The efficacy assessment of a diagnostic model for the early assessment of prognosis for secondary HLH treatment based on the above mentioned 13 CNV-related variables (ROC curve).

Multiple treatments have been proven to be effective for specific subtypes of HLH, but the outcomes of each subtype vary broadly among individuals. Further analyses were required to investigate the associations between CNVs and microbial community with treatment processes and outcomes of different HLH subtypes, particularly two infection-induced subtypes, EBV-HLH and nEBV-HLH. Based on the treatment outcomes, all cases were categorized into remission and non-remission groups. The positive rate of CNV occurrence in patients with different outcome was presented in **Figure 5**. Results showed that most samples from remission group (61/79) appeared to be CNV-negative, whereas in non-remission group, there was a fifty-fifty split between CNV-positive (10/20) and CNV-negative (10/20) samples (**Figure 5A**). It is suggested that samples from non-remission group appear to have higher CNV positive rate at general level, which was also observed in the EBV-HLH subtype and the M-HLH subtype (**Figure 5B**). The distribution of CNV on different chromosomes are distinct between remission and non-remission groups (**Supplementary Figure 6A**). Remission group showed a common amplification on chromosome 1, with frequency more than 40%. However, the most common alternation in non-remission group was deletion of DNA fragment on chromosome 19. These findings suggested that CNV distribution also leads to different treatment outcomes.

For association analysis between blood microbiome with treatment outcome, two parameters were described, the number of all microbes detected in each sample and the total RPM value of all microbes in each sample (**Figure 5**). Microbes were clustered into two groups determined by their etiological roles, as pathogens and non-pathogenic microbes. The microbe number (*P*=0.009) and the total RPM value (*P*=0.001) of pathogens in each sample showed significant difference between remission and non-remission groups, which indicated the association of the diversity and abundance of pathogenic microbes with treatment outcomes (**Figure 5C**). Whereas, both the number (*P*=0.420) and the total RPM value (*P*=0.560) of non-pathogenic microbes between remission and non-remission groups showed no significant difference (**Supplementary Figure 6B**). The landscape of microbial community of remission group and non-remission group in EBV-HLH subtype showed distinct patterns, with significant differences in the number of non-pathogenic microorganisms (*P*=0.009) (**Supplementary Figure 7**). Same analysis strategy was applied for nEBV-HLH subtype by comparing remission group and non-remission group at pathogen and non-pathogenic microbe level, but the results did not show consistent trend as in EBV-HLH subtype (**Supplementary Figure 8**). For EBV-HLH subtype, besides EBV infection being the most contribute pathogen, there are other co-infected pathogen existed, but appear no significant association with treatment outcomes (**Supplementary Figure 6C**). However, the heatmap of the overall microbial community illustrated the enrichment of bacteria and fungi in non-remission group, demonstrated the important role of non-virus species in therapy feedback of nEBV-HLH subtype (**Figure 5D**).

Although the above studies elevated CNVs and characteristic microorganisms as key parameters for early predictive assessment of secondary HLH clinical outcome, we found that perhaps only CNV-related key parameters could be used for early prognostic assessment of secondary HLH treatment when we constructed a Random Forest binary classifier model based on the above mentioned biomarkers to differentiate between secondary HLH treatment effects (R vs. NR). When we set the ratio of training and test sets to 3:1 and used the tenfold cross-validation method to determine the number of significant variables for classification, the optimal point of cross-validation error determined by the number of biomarkers was 13, which implies that based on the mean decreasing accuracy, the top 13 variables could be selected as potential markers used to differentiate between secondary HLH treatment effects (R vs. NR) (**Figure 5E**). Interestingly, the top 13 potential markers mentioned above were all CNV-related parameters, suggesting that changes in patients’ peripheral blood CNV may be closely related to the prognosis of secondary HLH treatment (**Figure 5E**). Finally, we gained further insight into the diagnostic performance of the binary classifier based on the above 13 biomarkers through ROC curves (**Figure 5F**). The results showed that the classifier model performed better in the early assessment of prognosis for secondary HLH treatment with an AUC value of 0.750 (**Figure 5F**).

## Discussion

Early diagnosis and appropriate targeted therapy may be an effective way to improve the survival and prognosis of patients with HLH. In clinical practice, patients with secondary HLH are often unclear at the initial diagnosis whether they have insidious diseases, such as complicated infections, tumours or autoimmune diseases, which can be important triggers of secondary HLH (26, 27) and are important directions for etiological screening, thus a comprehensive assessment and systematic diagnosis of patients with suspected HLH are needed. mNGS is commonly used for infectious pathogen detection and has also been proved promising in diagnosing various tumors including CNS malignancies, lung cancers and haematological malignancies (20, 21, 23, 24, 28, 29). In this study, we identified complex infections, tumours or autoimmune diseases by Onco-mNGS and immunological assay data, which were used as important parameters for constructing the Random Forest classifier model, performed key parameter screening to evaluate the effectiveness of the classifier in following study, and explored the novel Onco-mNGS in diagnosing secondary HLH triggered by different underlying diseases clinical value. The CNV and microbial data simultaneously generated by Onco-mNGS technology was further explored to identify a series of novel biomarkers that were available for typing different HLH subtypes. Based on these data, the treatment outcomes of HLH patients with different HLH subtypes were evaluated, which could be of potentially important clinical significance in accessing prognosis of HLH patients.

HLH is a rare, complicated and multifaceted syndrome that maybe triggered by various infectious agents, malignancies and rheumatologic disorders (30). The diagnosis of HLH is often delayed and misdiagnosis remains a significant concern due to the complexity of diagnostic criteria and similarity to other inflammatory disorders. Additionally, it is of vital importance to identify the secondary triggers to select the appropriate therapeutic strategies. In this study, 92 patients of secondary HLH were finally classified into four subtypes based on Onco-mNGS technology, including EBV or other pathogens triggered HLH, malignant associated HLH and rheumatological disease related HLH. Most of these patients in our study were triggered by infections and were predominantly associated with EBV. A regional study on adult HLH patients in China revealed that malignancy was the most common underlying cause, accounting for 42% of the cases, but EBV was a predominant pathogen in infection related HLH patients (31). However, the overall epidemiological investigation of HLH in China reported that EBV-HLH was the most common subtype, which may be related to the prevalence of EBV in our country (32). In this study, infection-induced HLH patients were further subdivided into EBV-associated and other pathogens-associated subtypes based on Onco-mNGS, which may better differentiate patients with different causative agents and contribute to precise anti-infection therapy. Our study indicated that Onco-mNGS reached a sensitivity of 60% in detecting patients with HLH associated with other infections (non-EBV), although it was consistent with conventional methods in patients with EBV-HLH, and therefore it may be served as a complement to conventional methods.

Genomic instability is a hallmark of cancer, and a large number of genomic alterations are present in human cancers, with aneuploidy (imbalance at the chromosome level) being one of the most common alterations (33, 34). Copy number variants (CNVs), as an indicative parameter of aneuploidy, are usually closely associated with the onset and progression of tumours and their associated diseases, and their effects often extend from the genomic level to the level of gene expression, ultimately giving rise to clinical phenotypes that are closely related to tumours and their associated diseases (35, 36). Generally, copy number variants (CNVs) in cancer patients may include deletion and duplication of small fragments, reduction/single nucleotide variants, gain or loss of chromosomal arms, or even doubling of entire chromosomes and whole genomes (37). CNV, as a comprehensive signalling parameter, consists of different variants from the genomic to the chromosomal level and involves copy number variation on different chromosomes, in particular whether copy number variation occurs on each chromosome, the type of copy number variation (deletions or duplications), and the degree of copy number variation (minor or major). In the current study, rapid identification of chromosomal CNVs including deletion and amplification were obtained based on the novel Onco-mNGS technology, and different forms (major and minor) within the two patterns represented changes in whole or focal sites of the chromosomes. Evidence have demonstrated that the prediction of aneuploidy-based tumor by mNGS not only expands the typical application scenarios of conventional mNGS for identifying potentially infectious agents, but also yields satisfactory performance in predicting malignancies, thus improving the diagnostic efficiency of malignant tumors (20, 21, 23, 24). CNV was observed in both patients with infection-induced HLH and patients with malignancy -associated HLH in our study but appeared to present distinct characteristics between the two subtypes. Compared with the EBV-HLH subtype, the M-HLH subtype had a higher incidence of CNVs on almost all chromosomes except chromosome 21, which represented frequent genomic instability. Besides, significantly different CNV types were found on chromosome 8, 13, 16, 18 and 19 between the two subtypes. These differences may be related to the different subtypes and their associated tumors, yet further studies are needed to explore the underlying causes and significance. When analysing the etiology of HLH, especially when constructing a classification model using the Random Forest classifier, it is often necessary to prefer high-performance parameters from a large number of subdivided parameters in order to obtain better classification results, and thus subdivided CNV parameters (e.g., which chromosome undergoes a CNV, the type of CNV, and the intensity of the CNV, etc.) are more valuable to analyse than a single parameter based solely on the presence or absence of a CNV. In fact, in this study, we constructed a random forest binary classification model based on biomarkers such as CNV and characteristic microorganisms to differentiate secondary HLH treatment effects (R vs. NR). When we set the ratio of training and test sets to 3:1 and used the tenfold cross-validation method to determine the number of significant variables for classification, the optimal point of cross-validation error determined by the number of biomarkers was 13, which implies that based on the mean decreasing accuracy, the top 13 variables could be selected as potential markers used to differentiate between secondary HLH treatment effects (R vs. NR) (**Figure 5E**). Interestingly, the top 13 potential markers mentioned above were all CNV-related parameters, suggesting that changes in patients’ peripheral blood CNV may be closely related to the prognosis of secondary HLH treatment (**Figure 5E**). Acute EBV infection can idiosyncratically lead to non-neoplastic HLH in patients without genetic predisposition (i.e. secondary HLH), while EBV-associated T/natural killer (NK)-cell lymphoproliferative disorders and lymphomas can induce neoplasia-associated HLH (38). As we known, T and NK cell lymphomas are the most dominant malignancies that induce HLH, and they are frequently associated with EBV infection (39). Therefore, it is believed that EBV infection plays an important role in the pathogenesis of HLH, which reminds us to carefully identify the complex etiology of patients with HLH in order to take appropriate therapeutic strategies and improve the prognosis of patients.

In some cases, infection and malignancy often co-exist in patients with HLH, and it is possible that HLH develops in association with triggering infections that occur as the result of chemotherapy-induced immunosuppression (40). Thus, both malignancy and infection may contribute to the cause of HLH in such situation (41). In fact, HLH patients have a high incidence of complicated infections and infection-related mortality due to the presence of multiple infection-related risks such as abnormal autoimmune status, combination chemotherapy, hemocytopenia, and the use of immunosuppressive drugs (42). In this study, we described the responsible pathogens that may cause infections in blood samples from patients with different HLH subtypes. The current study based on onco-mNGS have found that in addition to identifying such pathogens as EBV, CMV and *Staphylococcus aureus*, which are relatively common in bloodstream infections, other potential pathogens including *human alpha herpesvirus 1* and *Torque teno virus*, *human beta herpesvirus 6B* and *human alpha herpesvirus 3* were also detected. Previous studies have shown that the most commonly Gram-positive bacteria detected by mNGS were Staphylococcus aureus in blood samples from patients with diagnosed with sepsis and bloodstream infection, while human herpesvirus type 5 and Epstein-Barr virus were the most commonly detected viruses, which is consistent with our study (42). Notably, CMV was present in almost all subtypes of patients enrolled in this study, and this may be related to the generally lower immunocompetence of these HLH patients. In addition, mNGS was able to identify viral pathogens among patients with hematologic malignancies who developed sepsis (43). Bloodstream infection and sepsis remains a common but fatal complication among patients with immune suppression. Thus, complex infection in HLH is a serious and challenging disease that requires vigilance, early identification, and timely anti-infective therapy (40).

The human microbiome comprises a vast corpus of bacterial, archaeal, viral and fungal microbial taxa and microecology is increasingly considered to be involved in the onset and progression of diseases. It was hypothesized that the blood microbiome originates from the skin–oral–gut axis (44). A various of studies have described the blood microbial communities and evaluated the potential of blood microbiome dysbiosis as a prognostic marker in cardiovascular diseases, cirrhosis, severe acute pancreatitis, type 2 diabetes, and chronic kidney diseases (45–48). Blood microbiomes of HLH patients were also characterized based on our Onco-mNGS technology, and it was found that much lower microbial diversity was observed in patients with EBV-HLH than that in patients with other HLH subtypes due to anti-infective treatment. Several key microbial species enriched in the different subtypes were also identified, including *Streptococcus oralis*, *Rothia dentocariosa*, *Pseudomonas poae*, *Human alphaherpesvirus 3*, *Human betaherpesvirus 5* and *Bacillus cereus group*, *Human gammaherpesvirus 4*, and they may be responsible for the distinct microbial community among different HLH subtypes. Our results extend the knowledge of the blood microbiome in patients with HLH.

It is difficult to diagnose and initiate treatment in patients with secondary HLH because of the overlap of the signs and symptoms with a wide range of chronic conditions, including sepsis, multiple organ dysfunction, malignancy, or progression of rheumatic diseases (49, 50). Many biomarkers have been proposed for the diagnosis of HLH in both adults and children. Previous studies have established diagnostic models for differential diagnosis of secondary HLH by evaluating a serial of cytokine levels and HScore, which have achieved a good diagnostic performance (51–53). However, the HScore was used for estimating an individual’s risk of having reactive hemophagocytic syndrome, not for the differential diagnosis of HLH or other underlying diseases (54, 55). For patients with secondary HLH, good medical management and follow-up depend on the underlying trigger of HLH in these patients. Since we have defined the distinct characteristics of different HLH subtypes on CNV profile, infectious pathogen spectrum and blood microbial community, a random forest classification model was thereby developed based on these data which allowed us to better identify the different HLH subtypes and determine the main triggers. According to the current classification model, a total of four variables were included in this study, including *Human gammaherpesvirus 4*, *Human polyomavirus 1*, WBC and *Torque teno virus*. It can make timely and precise classifications among different HLH subtypes through applying this predictive model, which is beneficial to guide further targeted treatment.

However, even with the prompt administration of specific therapeutic strategy, treatment response and overall survival rates of HLH patients remain markedly worse, especially when the condition is associated with malignancy and infection (56, 57). Poor prognosis were common not only in patients with malignancy-associated HLH, but also in those with active EBV infection and in some high-risk HLH patients of unknown cause, and many of them died of rapid deterioration due to severe sepsis and multi-organ failure (58–61). It is therefore of great importance for clinicians to identify high-risk patients earlier in the course of management. Few studies have been conducted to identify factors associated with treatment outcomes in patients with secondary HLH based on CNV and microbiological data. The current results have selected a serial of predictive factors to identify high-risk patients with poor treatment outcomes. Our study showed that patients with positive CNV results and frequent deletions of DNA fragment on chromosome 19 were more likely associated with unfavorable treatment outcomes. Considering microbial factors, it was found that poor treatment outcomes were also associated with patients with higher pathogen burdens. Meanwhile, a disturbed blood microbiome, especially enriched bacteria and fungi, was related with a worse response to treatment. The treatment outcomes of different HLH subtypes were also investigated. In the EBV-HLH, lower non-pathogenic microbes were found to be significant correlated with poor outcomes. Combinations of these predictive factors may allow clinicians to identify patients at high risk of poor prognosis and more quickly adapt the therapeutic strategy, although a larger sample, multicenter, randomized controlled clinical cohort is needed to further study and verify. In fact, although the random forest classifier model established in this study showed relatively good working efficacy in the stratified diagnosis of secondary HLH etiology and early prediction of treatment prognosis, the overall small sample size caused by multiple factors is still an important drawback of this study, including the low prevalence of HLH (about 1-225/300,000 in children and 1/2000 in adults), the difficulty of definitive diagnosis of secondary HLH etiology based on the existing clinical diagnostic conditions, and the difficulty of tracking patient information due to the relatively complex and lengthy consultation process. We believe that based on the technical platform and research paradigm established in this study, as well as the preliminary results obtained, the subsequent establishment of a multi-geographical and multi-centre study cohort with a large sample size will further optimize and improve the composition of the relevant biomarker set, and significantly enhance the effectiveness of the Random Forest classifier model in the etiological diagnosis of secondary HLH and the early prediction of the prognosis of treatment.

In conclusion, our study demonstrated that the novel Onco-mNGS is able to identify the infection and malignancy- related triggers among patients with secondary HLH. A random forest classification model based on CNV profile, infectious pathogen spectrum and blood microbial community was developed to better identify the different HLH subtypes and determine the underlying triggers. The prognosis for treatment of HLH patients is not only associated with CNV, but also with the presence of pathogens and non- pathogens in peripheral blood. Higher CNV burden along with frequent deletions on chromosome 19, higher pathogen burden and lower non-pathogenic microbes were prognosis factors that significantly related with unfavorable treatment outcomes. Our study provided comprehensive knowledge in the triggers and prognostic predictors of patients with secondary HLH, which may help early diagnosis and appropriate targeted therapy, thus improving the survival and prognosis of the patients.

## Data Availability

The datasets presented in this study can be found in online repositories. The names of the repository/repositories and accession number(s) can be found below: PRJNA1072283 (SRA).

## Supplementary material

Supplementary Figure 1 The distribution of pathogens identified by mNGS in this study. **(A)** Heatmap of pathogens identified by mNGS sequencing. Log10-transformed RPM of selected microbes were applied. Samples were hierarchically clustered within each subtype using Pearson correlation as a distance measure with average-linkage. **(B)** The composition of pathogenic microbes in each subtype. All pathogens were adjudicated by clinical physicians. Log2-transformed RPM were applied for the analysis.

Supplementary Figure 2 Pathogen composition in each sample collected from patient with EBV-HLH (A), non-EBV-HLH (B), M-HLH (C) and Rh-HLH (D) subtype. All pathogens were adjudicated by clinical physicians. Log2-transformed RPM were applied for the analysis.

Supplementary Figure 3 Positive rate and diagnositic performance of mNGS. **(A)** Positive detection rate of mNGS in different HLH subtypes. **(B)** Taxonomic composition of microbes identified by mNGS in all blood samples. **(C)** Diagnostic performance of mNGS in identifying EBV-HLH subtypes (n=51) and nEBV-HLH subtypes (n=21) when compared with clinical gold standards. For identificaiton of EBV-HLH and nEBV-HLH subtype, sensitivity of mNGS was 86.7% and 100% respectively.

Supplementary Figure 4 Detection and burden of EBV and CMV in blood from patients with different HLH subtypes. **(A)** EBV and CMV positive rate detected by mNGS sequencing. **(B)** EBV and CMV burdens between different HLH subtypes. Differences between groups were assessed using T-test. Significant differences between groups are indicated by asterisks, with *** represents *P*<0.001. Abbreviations: EBV, *Epstein-Barr virus*; CMV, *Cytomegalovirus*; NS., no significant difference.

Supplementary Figure 5 Analysis of blood microbiome composition and diversity in HLH patients with different etiologies. **(A)** Heatmap of blood microorganisms with frequencies above 2% in each HLH subtype identified by mNGS. Log10-transformed RPM of selected microbes were applied. Samples were hierarchically clustered within each subtype using Pearson correlation as a distances measure with average-linkage. **(B)** Microbial alpha diversity based on species profile within each subtype was evaluated using shannon index, richness, simpson index and inverse simpson index. Differences between groups were assessed using anova. **(C)** Microbial beta diversity based on species profile between subtypes was evaluated through Unconstrained PCoA (for principal coordinates PCoA1 and PCoA2) by Bray–Curtis distance, with permutational multivariate analysis of variance (PERMANOVA) by Adonis.

Supplementary Figure 6 The association of treatment outcome in HLH patients with CNV characteristics and blood microbiome respectively. **(A)** Distribution of DNA fragment gain (amplification) or loss (deletion) in each chromosome in EBV-HLH and M-HLH patients with different treatment outcomes. Abbreviations: R, remission; NR, non-remission. **(B)** Comparison of the counts and burdens of non-pathogenic microbes in HLH patients with different treatment outcomes. Differences between groups were assessed using T-test. **(C)** Heatmap of co-infected microbes with frequencies above 5% in each group (R and NR) identified by mNGS in patients with EBV-HLH subtype. Log10-transformed RPM of co-infected microbes were applied. Samples were hierarchically clustered within each subtype using Pearson correlation as a distance measure with average-linkage.

Supplementary Figure 7 The association between blood microbiome and treatment outcome in EBV-HLH patients. **(A)** Comparison of the counts and burdens of pathogens in EBV-HLH patients with different treatment outcomes. Differences between groups were assessed using T-test. **(B)** Comparison of the counts and burdens of non-pathogenic microbes in EBV-HLH patients with different treatment outcomes. Differences between groups were assessed using T-test.

Supplementary Figure 8 The association between blood microbiome and treatment outcome in nEBV-HLH patients. **(A)** Comparison of the counts and burdens of pathogens in nEBV-HLH patients with different treatment outcomes. Differences between groups were assessed using T-test. **(B)** Comparison of the counts and burdens of non-pathogenic microbes in nEBV-HLH patients with different treatment outcomes. Differences between groups were assessed using T-test.
