# Supplementary figures and images for: Etiological stratification and prognostic assessment of haemophagocytic lymphohistiocytosis by machine learning on onco-mNGS data and clinical data
Source: Front Immunol. 2024 Sep 9;15:1390298. doi: 10.3389/fimmu.2024.1390298 (PMC11416948; doi:10.3389/fimmu.2024.1390298)

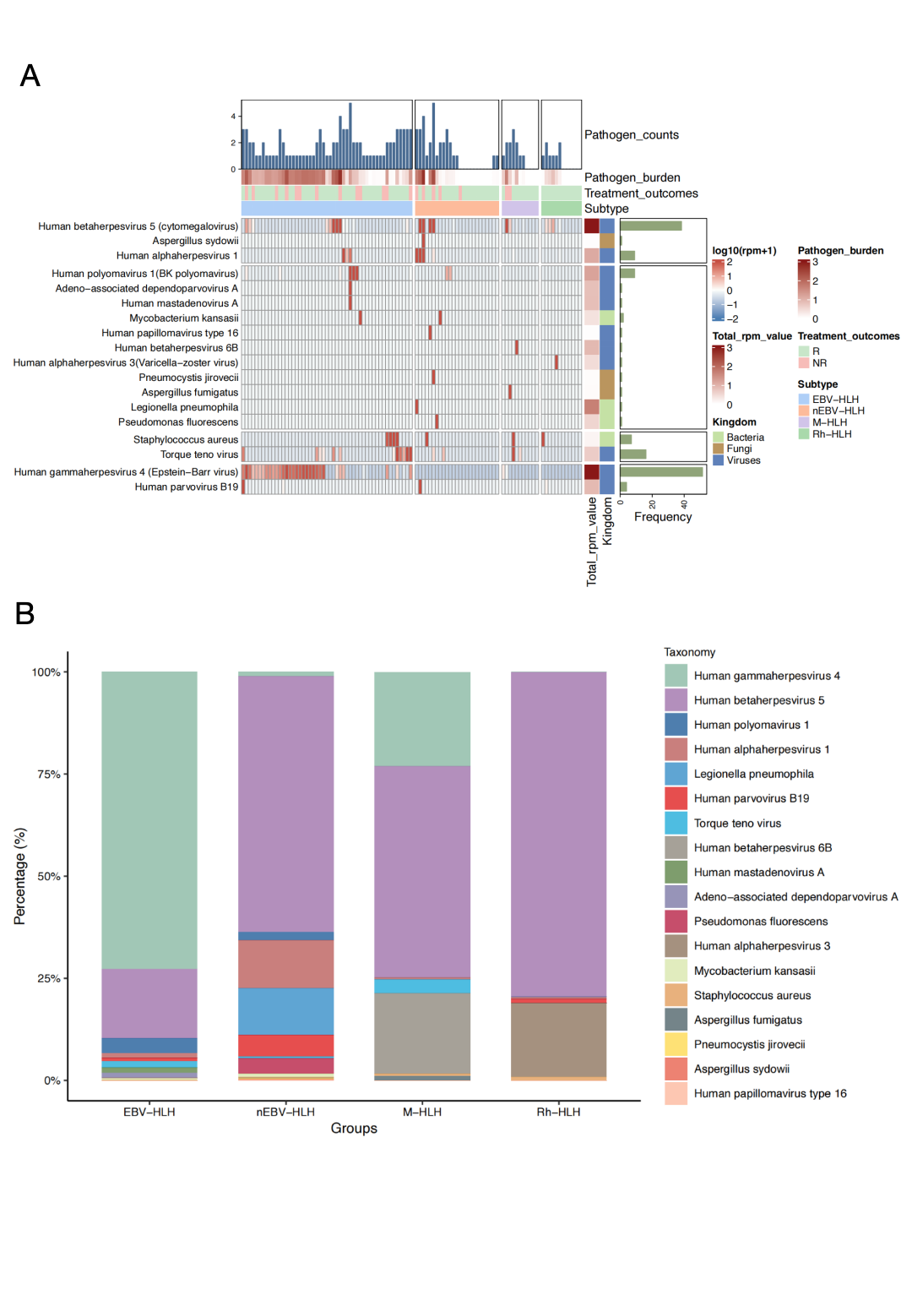

Supplement: Supplementary Figure 1 — The distribution of pathogens identified by mNGS in this study. (A) Heatmap of pathogens identified by mNGS sequencing. Log10-transformed RPM of selected microbes were applied. Samples were hierarchically clustered within each subtype using Pearson correlation as a distance measure with average-linkage. (B) The composition of pathogenic microbes in each subtype. All pathogens were adjudicated by clinical physicians. Log2-transformed RPM were applied for the analysis. [file Image1.tif]

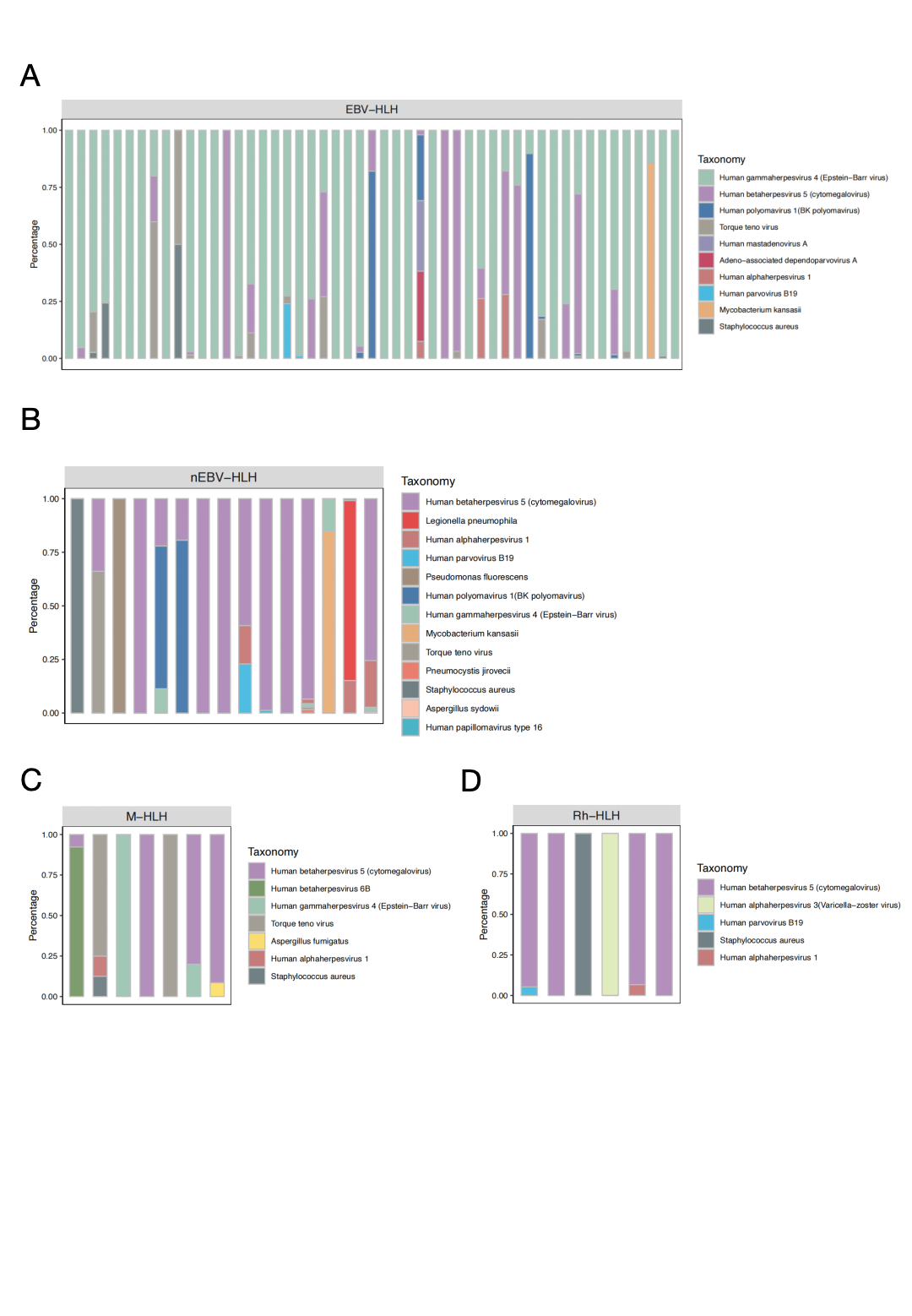

Supplement: Supplementary Figure 2 — Pathogen composition in each sample collected from patient with EBV-HLH (A), non-EBV-HLH (B), M-HLH (C) and Rh-HLH (D) subtype. All pathogens were adjudicated by clinical physicians. Log2-transformed RPM were applied for the analysis. [file Image2.tif]

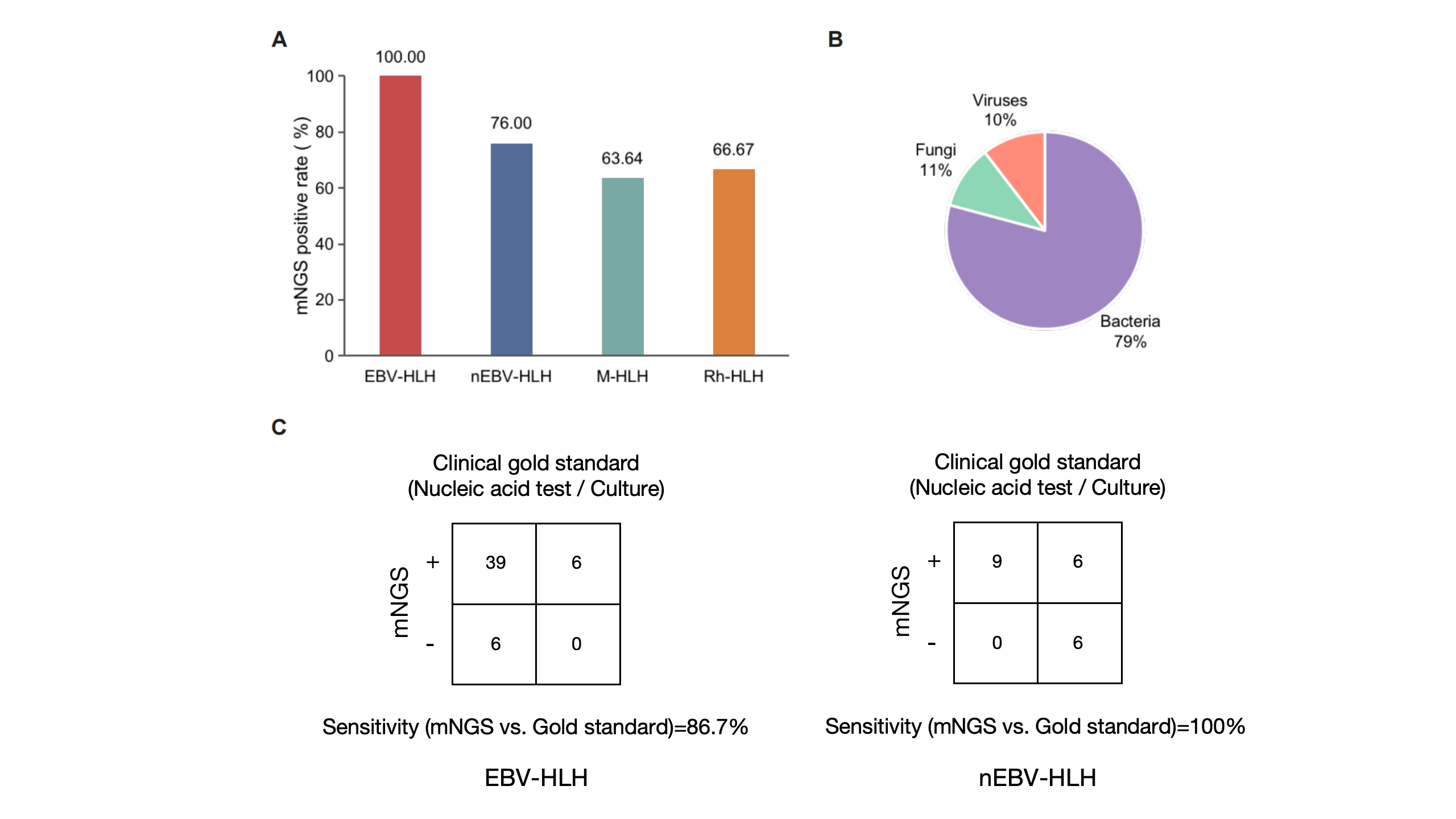

Supplement: Supplementary Figure 3 — Positive rate and diagnositic performance of mNGS. (A) Positive detection rate of mNGS in different HLH subtypes. (B) Taxonomic composition of microbes identified by mNGS in all blood samples. (C) Diagnostic performance of mNGS in identifying EBV-HLH subtypes (n=51) and nEBV-HLH subtypes (n=21) when compared with clinical gold standards. For identificaiton of EBV-HLH and nEBV-HLH subtype, sensitivity of mNGS was 86.7% and 100% respectively. [file Image3.tif]

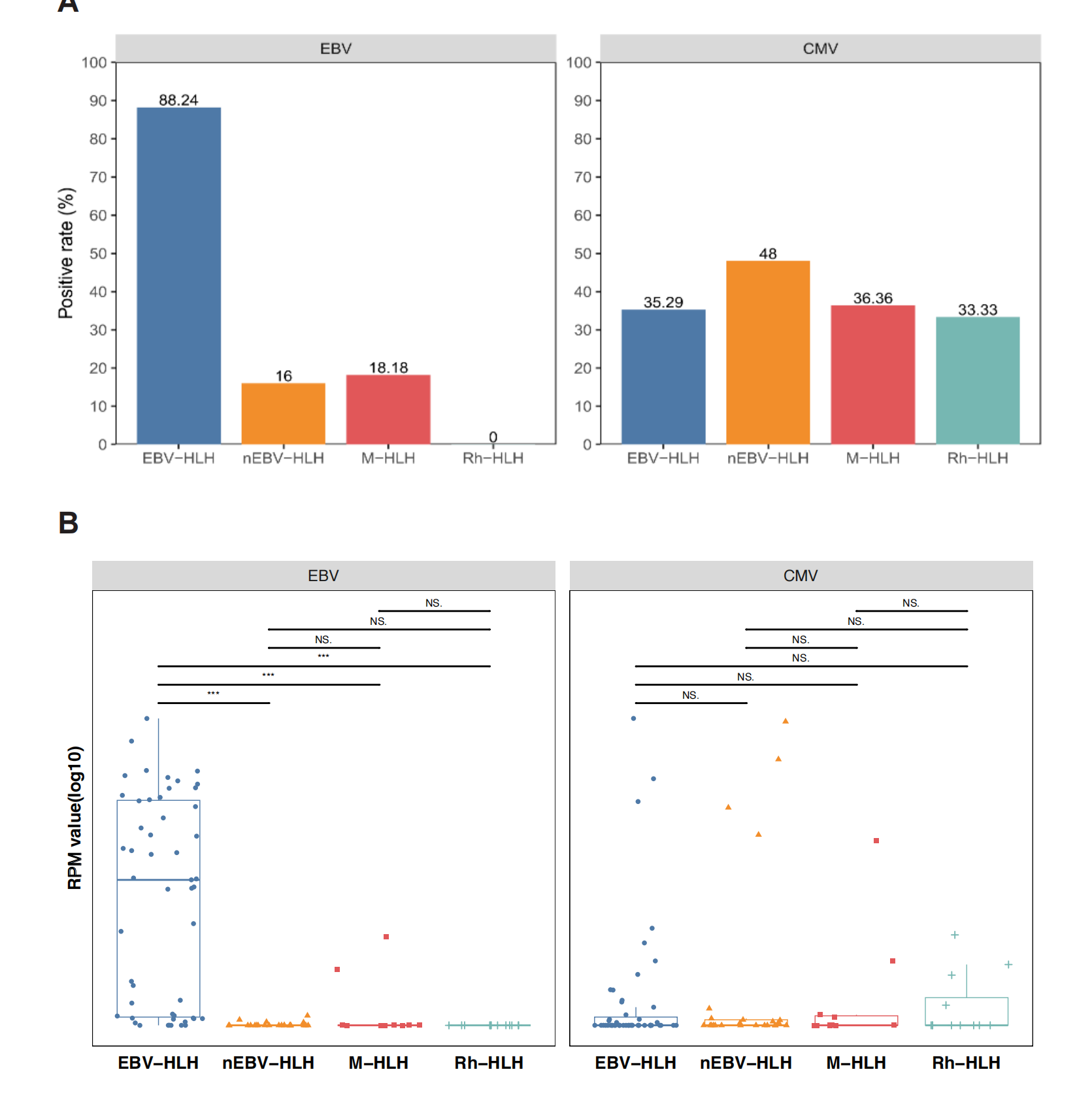

Supplement: Supplementary Figure 4 — Detection and burden of EBV and CMV in blood from patients with different HLH subtypes. (A) EBV and CMV positive rate detected by mNGS sequencing. (B) EBV and CMV burdens between different HLH subtypes. Differences between groups were assessed using T-test. Significant differences between groups are indicated by asterisks, with *** represents P<0.001. Abbreviations: EBV, Epstein-Barr virus; CMV, Cytomegalovirus; NS., no significant difference. [file Image4.tif]

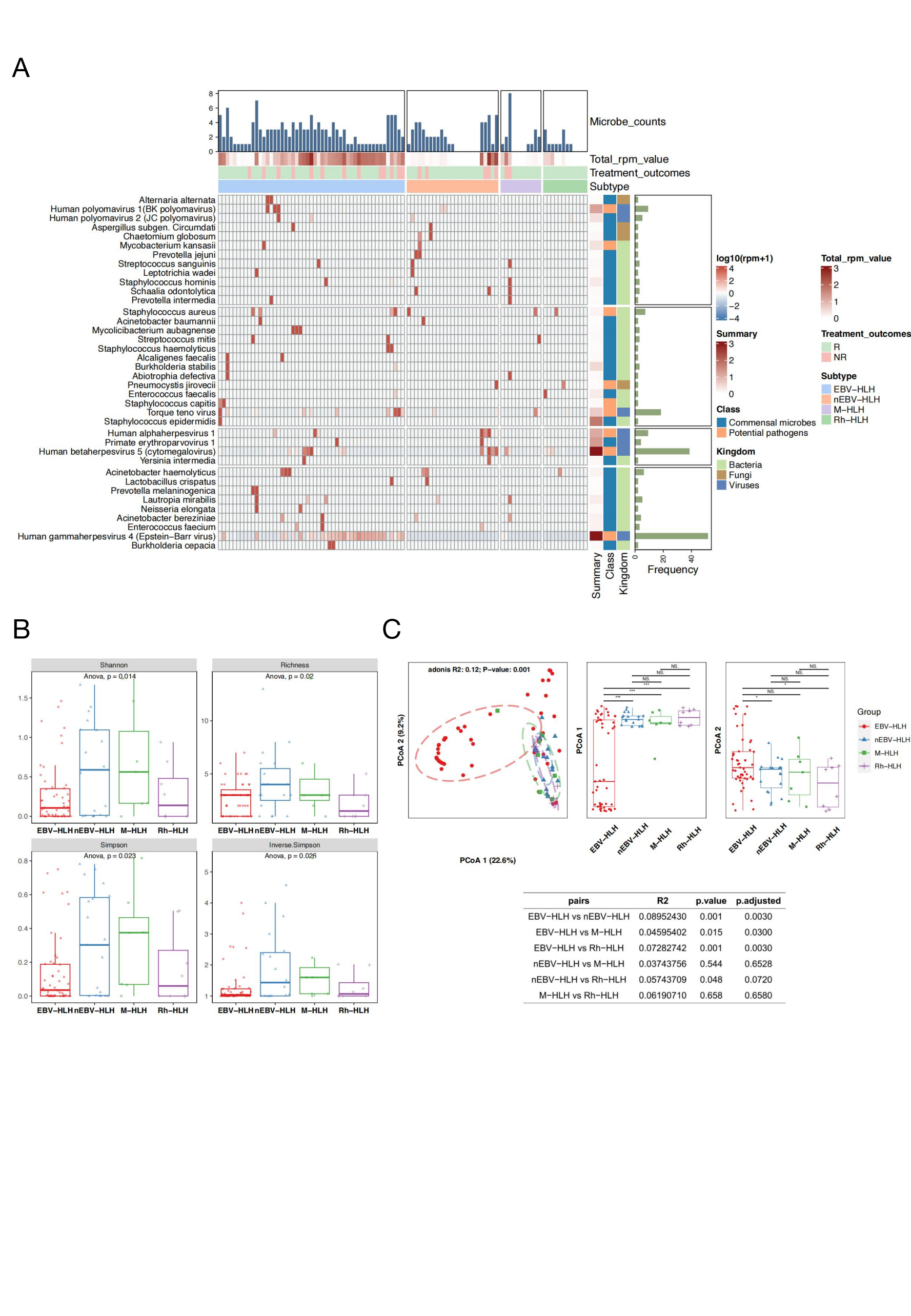

Supplement: Supplementary Figure 5 — Analysis of blood microbiome composition and diversity in HLH patients with different etiologies. (A) Heatmap of blood microorganisms with frequencies above 2% in each HLH subtype identified by mNGS. Log10-transformed RPM of selected microbes were applied. Samples were hierarchically clustered within each subtype using Pearson correlation as a distances measure with average-linkage. (B) Microbial alpha diversity based on species profile within each subtype was evaluated using shannon index, richness, simpson index and inverse simpson index. Differences between groups were assessed using anova. (C) Microbial beta diversity based on species profile between subtypes was evaluated through Unconstrained PCoA (for principal coordinates PCoA1 and PCoA2) by Bray–Curtis distance, with permutational multivariate analysis of variance (PERMANOVA) by Adonis. [file Image5.tif]

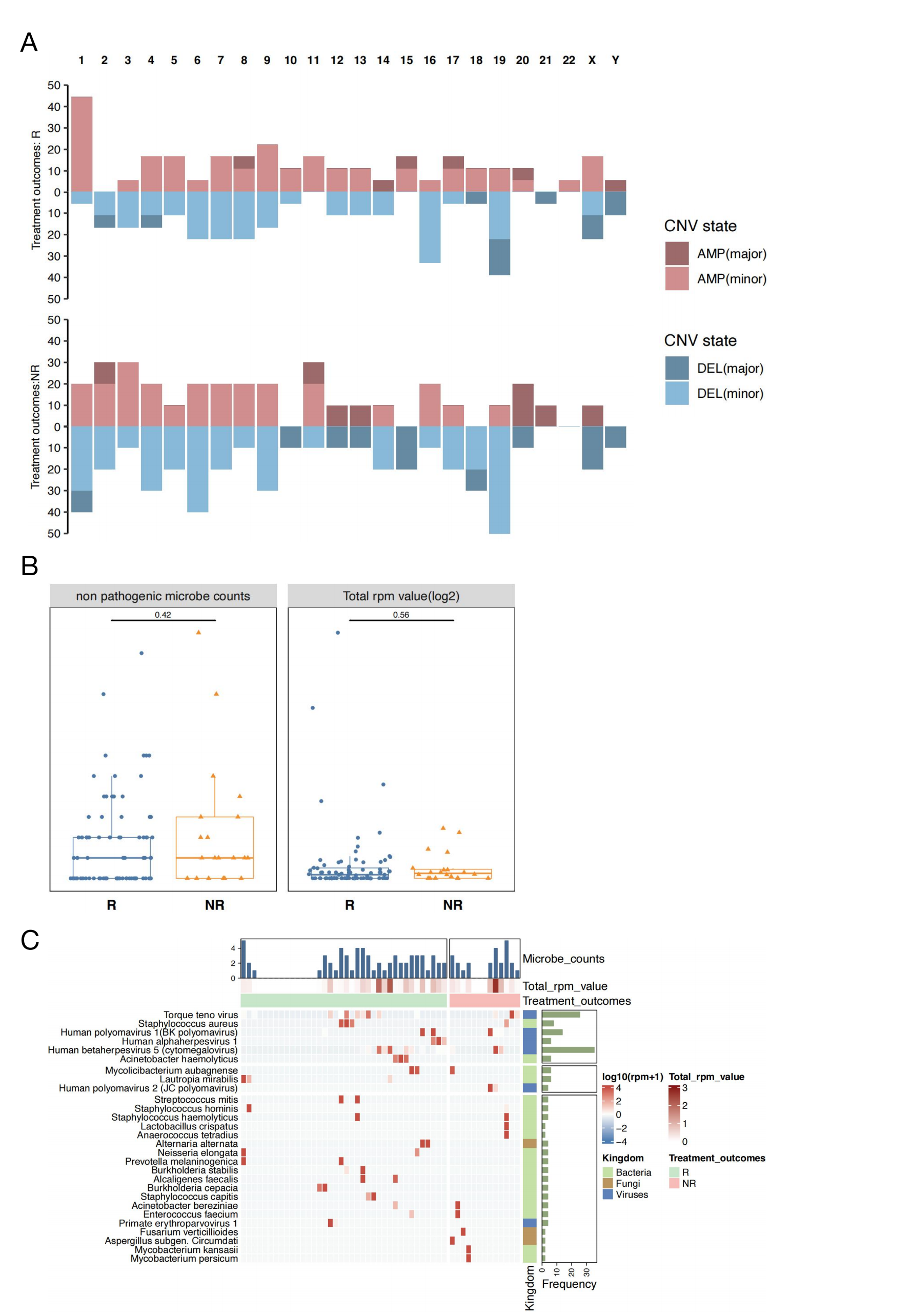

Supplement: Supplementary Figure 6 — The association of treatment outcome in HLH patients with CNV characteristics and blood microbiome respectively. (A) Distribution of DNA fragment gain (amplification) or loss (deletion) in each chromosome in EBV-HLH and M-HLH patients with different treatment outcomes. Abbreviations: R, remission; NR, non-remission. (B) Comparison of the counts and burdens of non-pathogenic microbes in HLH patients with different treatment outcomes. Differences between groups were assessed using T-test. (C) Heatmap of co-infected microbes with frequencies above 5% in each group (R and NR) identified by mNGS in patients with EBV-HLH subtype. Log10-transformed RPM of co-infected microbes were applied. Samples were hierarchically clustered within each subtype using Pearson correlation as a distance measure with average-linkage. [file Image6.tif]

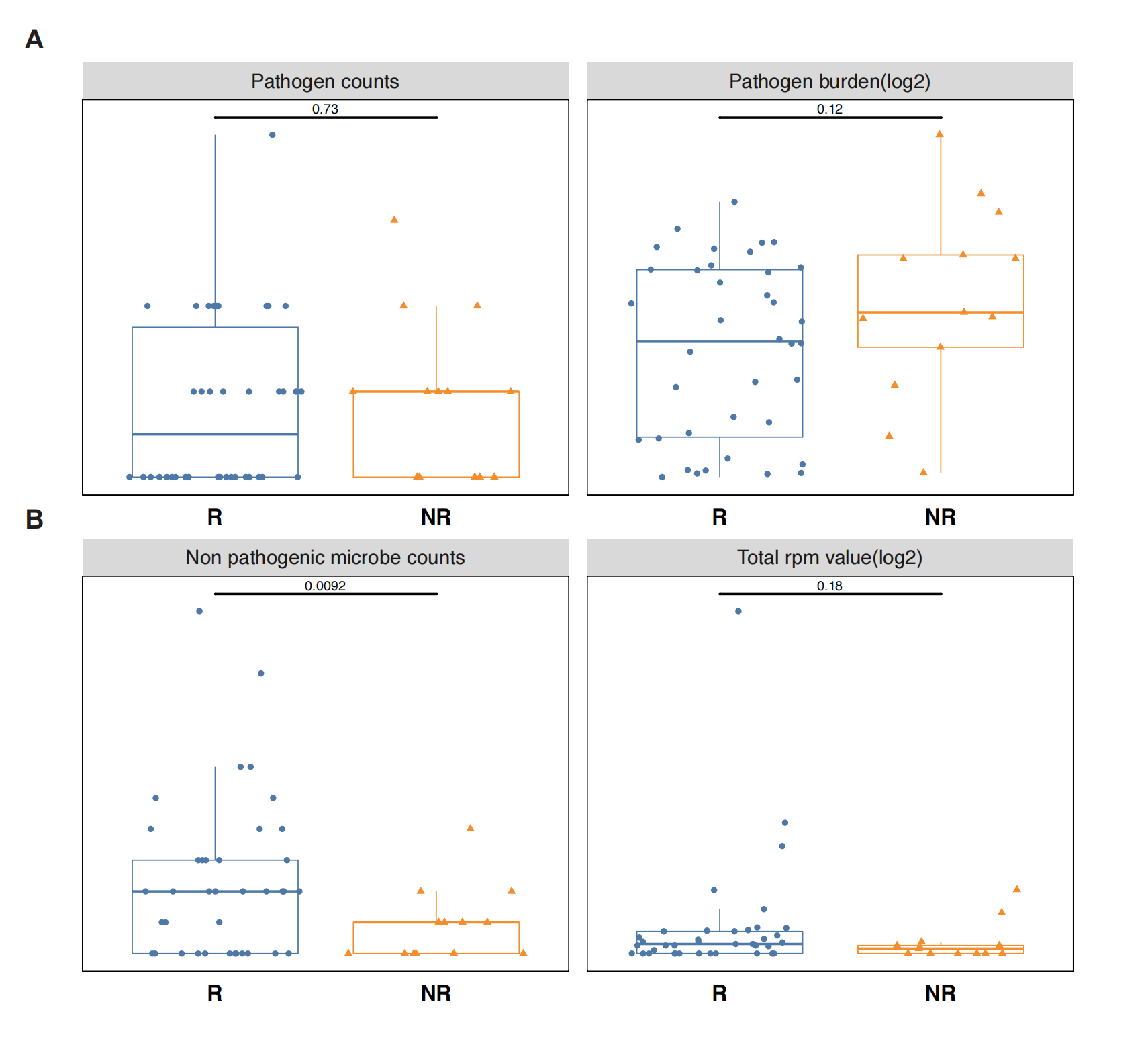

Supplement: Supplementary Figure 7 — The association between blood microbiome and treatment outcome in EBV-HLH patients. (A) Comparison of the counts and burdens of pathogens in EBV-HLH patients with different treatment outcomes. Differences between groups were assessed using T-test. (B) Comparison of the counts and burdens of non-pathogenic microbes in EBV-HLH patients with different treatment outcomes. Differences between groups were assessed using T-test. [file Image7.tif]

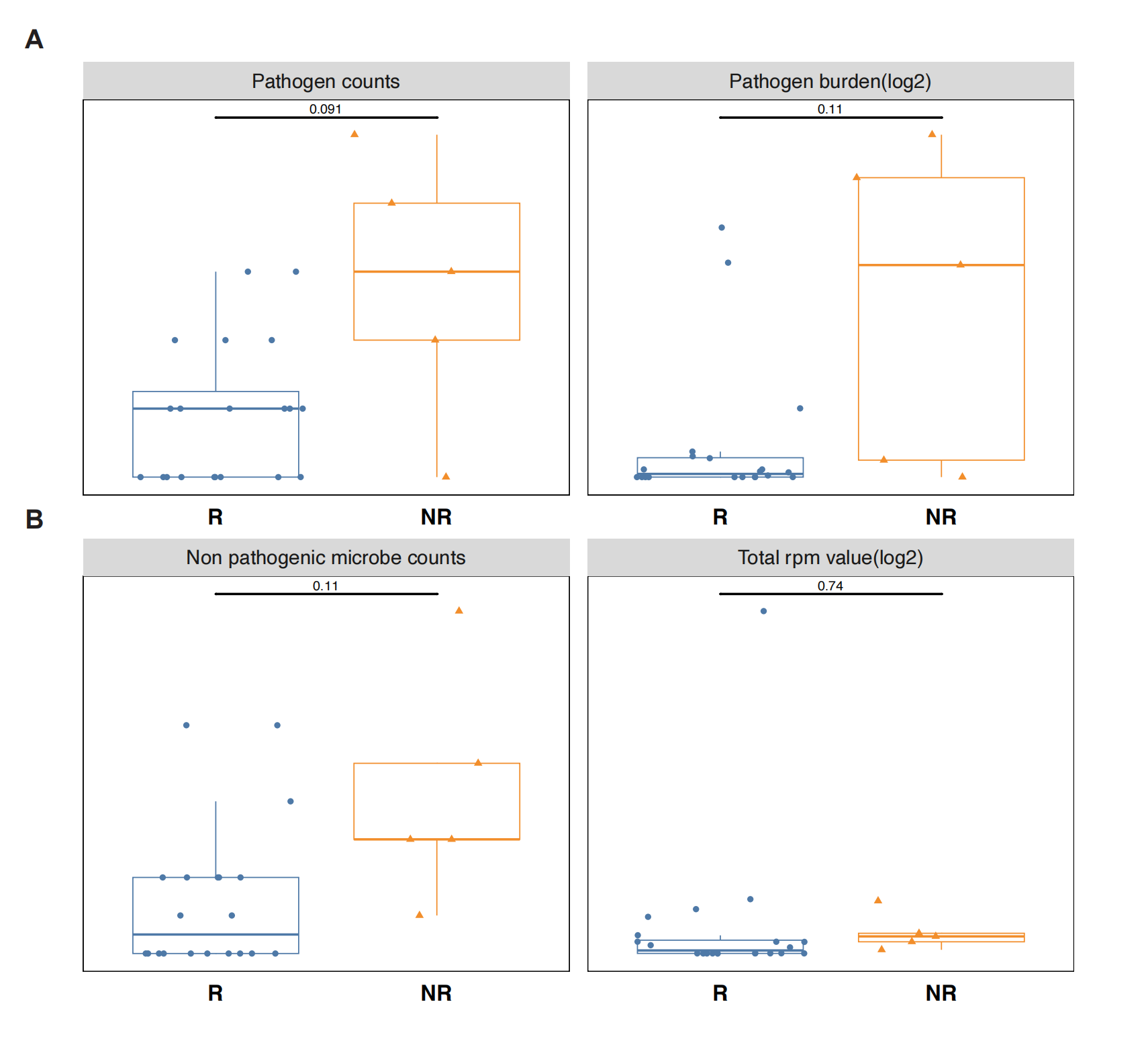

Supplement: Supplementary Figure 8 — The association between blood microbiome and treatment outcome in nEBV-HLH patients. (A) Comparison of the counts and burdens of pathogens in nEBV-HLH patients with different treatment outcomes. Differences between groups were assessed using T-test. (B) Comparison of the counts and burdens of non-pathogenic microbes in nEBV-HLH patients with different treatment outcomes. Differences between groups were assessed using T-test. [file Image8.tif]
